# Investigation of the human tear film proteome using multiple proteomic approaches

**Published:** 2008-03-07

**Authors:** Kari B. Green-Church, Kelly K. Nichols, Nan M. Kleinholz, Liwen Zhang, Jason J. Nichols

**Affiliations:** 1 Mass Spectrometry and Proteomics Facility; 2 College of Optometry, The Ohio State University, Columbus, OH

## Abstract

**Purpose:**

The purpose of this work was to examine the tear film proteome using a combination of one-dimensional (1D) and two dimensional (2D) gel electrophoresis and mass spectrometry-based techniques and to explore the effect of the tear collection methods on the tear proteome.

**Methods:**

Tear samples from eight normal non-contact lens wearing human subjects collected by Drummond glass microcapillary and Schirmer strips were subjected to 1D-sodium dodecyl sulfate polyacrylamide gel electrophoresis (SDS–PAGE), 2D-SDS–PAGE, and 2D LC-MS/MS (Multidimensional protein identification technology - MudPIT). Bands or cores from the 1D- and 2D-SDS–PAGE were cut, digested with trypsin, and analyzed by tandem mass spectrometry for identification by the generation of sequence tags.

**Results:**

In total (across sampling and proteomic methods), 97 unique proteins were observed, and a significant number of the spots/bands in the PAGE were from posttranslational modifications. Fifty-four unique proteins were identified from proteins extracted from the Schirmer strips in comparison to 13 unique proteins identified from capillary tubes, and 30 unique proteins were identified by both collection methods. Secreted (serum) proteins were predominantly observed from tears collected by capillary whereas a combination of cellular and serum proteins were identified from tear film collected by Schirmer strips.

**Conclusions:**

Overall, these results suggest that the tear film collection and the proteomic method impacts the proteins present in the tear film and that care should be exercised in choosing a tear collection method to best correlate to the experiment being conducted or the hypothesis that is being tested.

## Introduction

According to the annotated protein sequence derived from genome sequences, approximately 400,000 proteins have the potential to be expressed in the human alone. Many of these proteins are associated with normal human function and disease states [1]. Mass spectrometry (MS)-based proteomics possesses tremendous capabilities in the study of the entire differential output of proteins given the availability of genome sequence databases [1,2]. It also has several advantages over traditional methods such as chromatographic methods, electrophoretic methods, Edman degradation, immunological methods, and surface-enhanced laser desorption and ionization (SELDI). While SELDI, chromatographic, and gel-based methods alone can track the appearance, disappearance, or molecular weight shifts of proteins, they cannot identify proteins or measure the molecular weight (MW) of proteins with appreciable accuracy [3,4]. In addition, Edman degradation requires a large amount of sample and is ineffective on NH_2_-terminal blocked proteins [3,4]. ELISA and western blots can be somewhat presumptive relative to protein identification as they require the availability of a suitable and specific antibody. Further, SELDI-based methods are limited to low molecular weight proteins, typically less than 20 kDa. As such, these types of methods are more appropriate for screening samples after the species have been established via MS-based proteomics. The benefits of MS-based methods are numerous with routine sensitivity in the nanogram-picogram range, rapid speed of analysis, the ability to precisely and accurately determine protein identity, the ability to characterize modifications, and the ability to analyze protein expression levels [1,2]. This is true as MS allows for simultaneous accurate mass measures in addition to the determination of structural properties of molecules via tandem MS. Using traditional electrophoretic, liquid chromatographic, or new chromatographic methods such as multi-dimensional protein identification technology (MudPIT) [5,6] in conjunction with MS for protein identification provides the most complete view of a proteome distribution relative to charge (pI), molecular weight (MW), abundance, and interactions (i.e., protein–protein complex).

The current understanding of tear film proteomics, including differences in sampling techniques as well as a fundamental understanding of the core tear proteome, is limited in the literature. There is disagreement in the literature regarding the number of proteins in the tear film and the functions of the individual proteins. Some of these functions are thought to be protective relative to aiding in the ocular surface defense system (i.e., antimicrobial or inflammatory-related), related to ocular surface wound healing, or stability-promoting through interaction with other ligands (i.e., lipid-binding proteins). The up- or downregulation of these proteins may be indicative of disease mechanisms (i.e., dry eye disease). Tear film protein profiles have historically been characterized using gel electrophoresis and Edman degradation in which both have shown the major constituents to include lysozyme, lactoferrin, von Ebner’s gland protein (e.g., lipocalin and tear specific prealbumin), transferrin, serum albumin, secretory IgA, and lipophilin [7-12]. Using these methods, it was been estimated that 70%–85% of the total secretory protein can be accounted for by lipocalin, lysozyme, and lactoferrin [7,13]. However, many proteins go unidentified using these methods because they are either not detected (e.g., due to masking by high abundant proteins or low sensitivity), not separated within bands, or are NH_2_-terminally blocked and are identified by molecular weight and pI only. Sensitive immunoassay-based methods have identified other proteins to be present in the tear film of mammals including phospholipid transfer protein [14], growth factors [15-19], neurotrophic factors [20], cytokines [17,21-29], cell adhesion molecules [30], matrix metalloproteinases [25,31-33], bradykinins [34], tachykinins (e.g., substance P) [35,36], fibronectin [37], plasminogen activator [38], defensins, aquaporins [39], phospholipase [40], immunoglobulins [41], lactate dehydrogenase [42], and insulin [18]. Immunoassay-based methods can be superior when studying a specific or individual protein whereas mass spectrometry-based proteomics can examine thousands of proteins without the need for antibodies. Discoveries made by mass spectrometric methods can then be closely examined using immunoassay techniques for validation and clinical studies.

Although matrix-assisted laser desorption ionization (MALDI) time-of-flight (TOF) MS had been used to characterize low molecular weight protein masses [43], it was not until more recently that it and electrospray ionization (ESI) MS/MS were used to identify some novel species in the tears [12,13,44,45]. The list of proteins found associated with the tear film continues to grow, and one recent study reported “approximately 500 proteins were detected and unambiguously identified by LC/MS/MS” [46], although the protein identities were not provided by the authors. More recent MS-based methods have started to reveal other unique proteins in the tear film. De Souza and coworkers [6] recently published the identification of 491 proteins from the tear film using a hybrid linear trap, Fourier Transform (LTQ-FT), and a linear ion trap, orbitrap (LTQ-Orbitrap). Recently, Ham et al. [47] used MALDI-TOF to examine proteins from normal and dry eye model rabbits. Similarly, Zhou et al. [12] analyzed rabbit tears using HPLC and electrospray ionization. However, while many proteins are commonly observed across these MS-based studies (i.e., lysozyme, lactoferrin, lipocalin, etc), many proteins appear to be unique to the study and may be associated with specific methodologies. Thus, the aims of this work were to examine the tear film proteome using a combination of one-dimensional (1D) and two-dimensional (2D) gel electrophoresis and mass spectrometry-based proteomics and to evaluate the differences in collection techniques on the measured tear proteome.

## Methods

**Figure 1:**
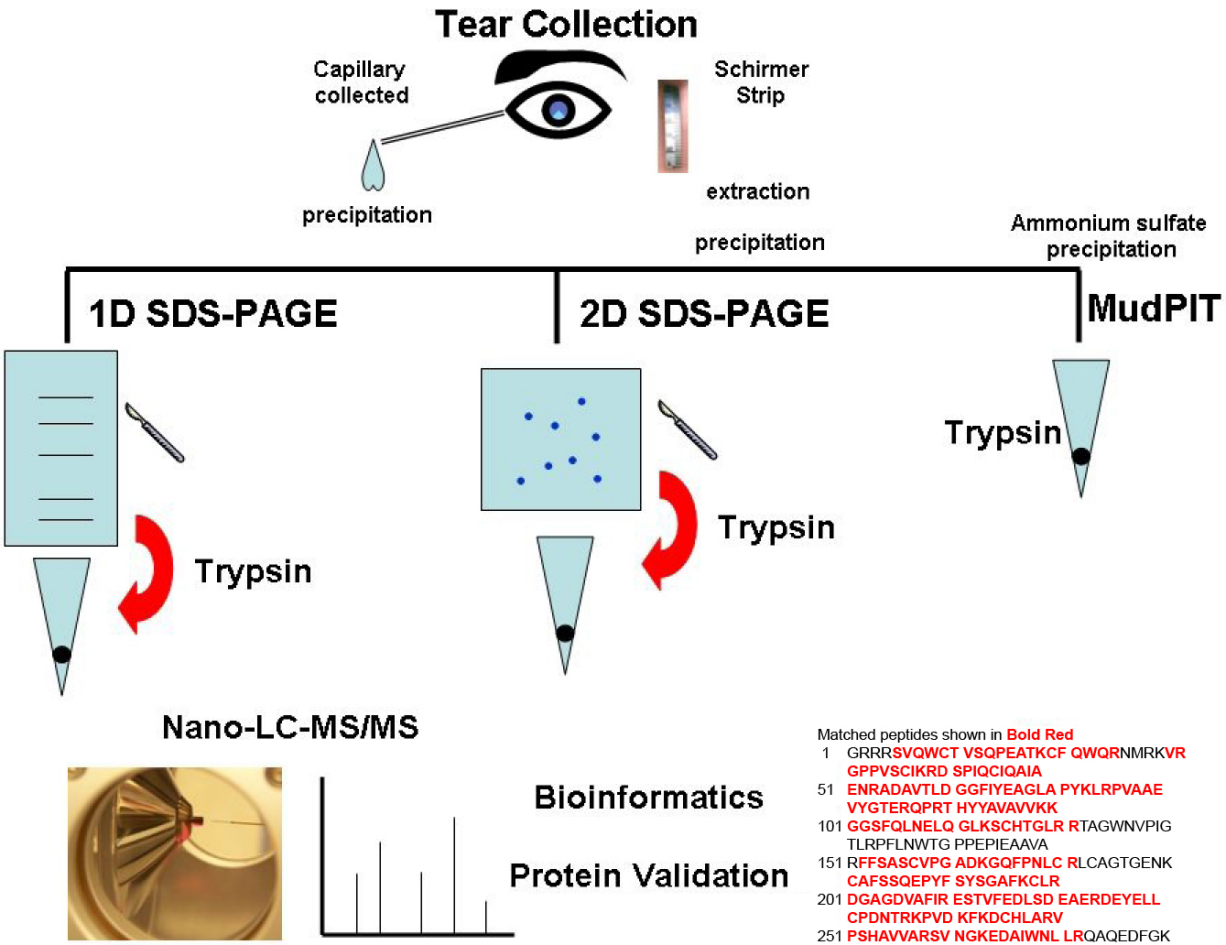
Work flow of the methods used in this manuscript. Tears were collected by capillary or Schirmer strips. The proteins were extracted using acetone precipitation and then were subject to either 1D-SDS–PAGE or 2D-SDS–PAGE or were directly digested into peptides with trypsin.

Figure 1 is a work flow diagram of the experiments performed in this study. Generally, proteomic work flow includes protein purification to remove salts, lipids, and non-protein substances from the biofluid followed by protein separation (chromatography), mass spectrometry analysis, and finally, bioinformatics. The methods chosen here include protein precipitation to remove the non-protein substances from the samples, 1D or 2D SDS–PAGE for protein separation, and in-gel digestion of individual protein spots or bands with trypsin to produce small peptides for analysis on nano-LC-MS/MS for protein identification. The exception is the multidimensional protein identification technology (MudPIT) where proteins precipitated from tears or Schirmer strips were not separated with SDS–PAGE and instead the entire protein mixture is digested with trypsin and analyzed with 2D-LC-MS/MS.

### Tear sampling

Eight subjects were seen on multiple occasions for tear film sampling (average=8 visits, range 3–18 visits). The average age (± SD) of the patients was 35 ± 13 years (range 24–55 years). Six of the eight subjects were female (75%), and seven of the eight (88%) were Caucasian (one was African-American). All participants were normal with no ocular disease, using no current eye medications, and none had eye-related symptoms (by patient report).

Tears were collected using small volume (1–5 µl) Drummond glass microcapillary tubes under 16X slit-lamp magnification. Non-reflex tears were collected from the inferior tear prism without contact with the lower lid until a total of 5 µl had been collected. During a separate visit, tear collection was performed by placing a Schirmer strip over the lower lid. The lid was not anesthetized and the strip was placed approximately 6 mm from the lateral canthus. The subject was instructed to close his/her eyes for the 5 min test duration, the wet length was not recorded but was observed to be within normal ranges in all cases. The strip was then placed in a 1.6 ml amber Eppendorf tube at 4 °C until analysis. Gloves were worn by the examiner for both collection methods and by all investigators handling any tear film samples.

### Protein sample preparation and quantitation

**Figure 2:**
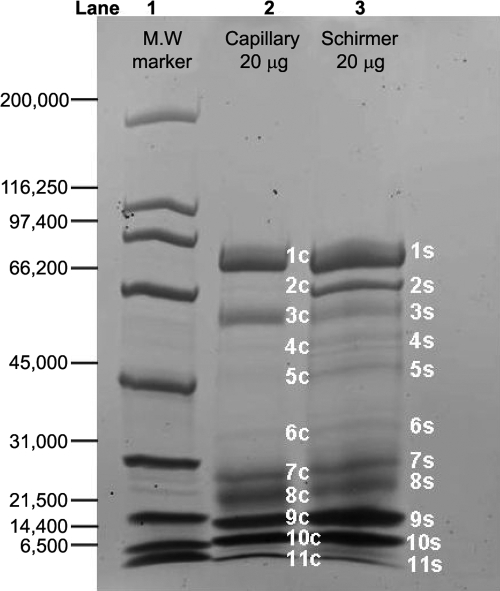
1D-SDS–PAGE of coomassie stained proteins. Lane 1 is the molecular weight marker. Lane 2 is 20 μg of total protein that was precipitated from tear film collected by capillary. Lane 3 is 20 μg of total protein that was precipitated from tear film collected by Schirmer strip. The observed bands are labeled 1−11.

Tear proteins collected by the capillary method were pooled from different patients and precipitated using acetone. The number of samples pooled for each individual experiment was dependent on the protein amount required and ranged from 3 to 16 pooled samples. Briefly, acetone was added at −20 °C at a volume four times that of the sample to be precipitated. The tube was vortexed and incubated for 60 min at −20 °C. The proteins were pelleted by centrifuging for 10 min at 13,000 xg. The acetone was removed leaving the protein pellet in the tube. Proteins collected by the Schirmer strip method were extracted by incubating the Schirmer strips in approximately 100 μl of 100 mM ammonium bicarbonate at room temperature for 1 h and then precipitating the solution as described above. Precipitated proteins were resuspended in a solubilization buffer (8 M Urea, 0.5% CHAPS) or in pure water. Proteins were quantitated by Bradford assay [48] using Coomassie plus protein assay reagent (Pierce, Rockford, IL) and BSA as a protein standard. The larger amounts of protein recovered from the Schirmer strip method sometimes made pooling unnecessary, although protein extracts from four Schirmer strips were typically pooled together for each experiment.

### 1D SDS–PAGE

Protein samples were mixed with SDS–PAGE loading buffer containing β-mercaptoethanol, heated to 95 °C for 5 min, and subjected to SDS–PAGE analysis using the Mini-Protean-III module (Bio-Rad, Hercules, CA). Gradient gels (4%–15% acrylamide) were pre-cast (Bio-Rad), and homogenous gels (10% and 18% acrylamide) were cast in-laboratory. Gels were fixed and stained with either Coomassie brilliant blue (Bio-Rad) or SyproRuby (Invitrogen, Carlsbad, CA) according to the manufacturer’s protocol. The gels were then imaged with a Typhoon 9400 variable mode scanner (GE Healthcare, Piscataway, NJ). ImageJ software (Rasband, W.S., ImageJ, USA National Institutes of Health, Bethesda, Maryland, 1997–2006) was used to examine lane profiles.

### 2D-SDS–PAGE mini gels

Protein samples (15 μg per gel) were diluted to 125 μl in a rehydration buffer (8 M urea, 0.5% CHAPS, 2.6 mg/ml dithiothreitol, 0.002% bromophenol blue, 0.5% pH 3–10 immobilized pH gradient (IPG) buffer; GE Healthcare, Piscataway NJ). Isoelectric focusing strips were focused on an IPGphor-II (GE Healthcare) with a four step IEF: the voltage was held at 300 V for 30 min, a gradient to 1000 V was applied for 30 min, a gradient to 5000 V was applied for 80 min, and lastly the 5000 V was held for approximately held for 15 min, and IEF strips were immediately used for SDS–PAGE. IEF strips were equilibrated with 5 ml of equilibration buffer 1 (6 M urea, 2% SDS, 29.3% glycerol, 0.002% bromophenol blue, 2.6 mg/ml dithiothreitol) for 15 min. IEF strips were then equilibrated in EQ buffer 2 (6 M urea, 2% SDS, 29.3% glycerol, 0.002% bromophenol blue, 6.5 mg/ml iodoacetamide) for 15 min. IEF strips were run on 16% acrylamide SDS–PAGE gels as described above.

### In-gel digestion

Protein spots were excised from the gel using a scalpel or a gel slicer. Trypsin was used to cut the protein into peptides by cleaving arginine and lysine residues to produce a searchable pattern of peptides. Individual bands or spots were digested with sequencing grade trypsin from Promega (Madison, WI) using the Montage In-Gel Digestion Kit from Millipore (Bedford, MA) following the manufacturers’ recommended protocols. The gels were washed in 50% methanol/5% acetic acid for 1–2 h. The gel bands were dried with acetonitrile and reconstituted with dithiothreitol (DTT) solution at 37 °C for 1 h to reduce cysteines. Iodoacetamide was added and incubated for 1 h at room temperature in the dark to alkylate cysteines. Trypsin was added and digested at room temperature overnight. The resulting peptides were extracted from the polyacrylamide gel with 50% acetonitrile and 5% formic acid several times and pooled together and concentrated in a speed vacuum to approximately 25 μl.

**Table 1:** Proteins identified by LC-MS/MS on a linear ion trap from tear film and analyzed by 1D-SDS–PAGE.

**Identification from Schirmer strip extracted proteins in band 4**	**Mowse score**	**Number of peptides**	**% sequence coverage**
gi|187122 Lactoferrin	5797	44	66
gi|13325287 Enolase 1	1931	25	68
gi|23241675 Serum Albumin	1098	23	45
gi|38026 Zn-alpha2-glycoprotein	1026	18	51
gi|37046835 Proline rich 4	575	2	16
gi|2183299 Aldehyde dehydrogenase 1	435	10	25
gi|27270813 IGHM protein	407	11	34
gi|113584 Ig alpha-1	386	7	33
gi|16306550 selenium binding protein	361	11	32
gi|306882 Haptoglobin	174	7	17
**Identification from capillary collected proteins in band 4**	**Mowse score**	**Number of peptides**	**% sequence coverage**
gi|187122 Lactoferrin	4954	45	70
gi|113584 Ig alpha-1	611	11	51
gi|38026 Zn-alpha2-glycoprotein	591	13	52
gi|4504963 Lipocalin 1	186	5	28
gi|31377806 Poly Ig Receptor	143	5	7
gi|623409 Keratin 10	117	2	3
gi|47132620 Keratin 2	80	2	3

Tear film proteins were collected by Schirmer strip or by capillary. The chromatography bands are those associated with Figure 1.

**Figure 3:**
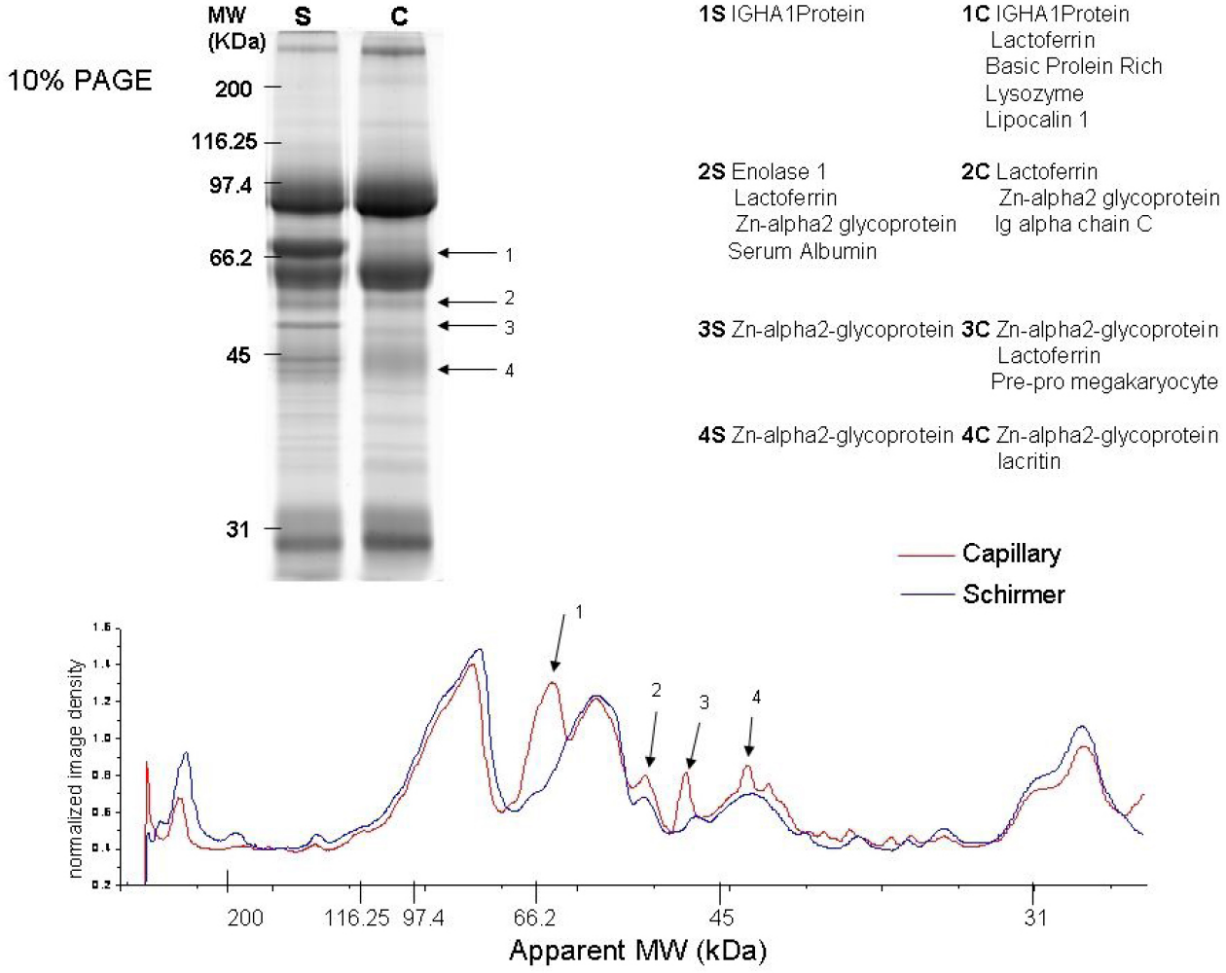
10% 1D-SDS–PAGE of coomassie stained proteins optimized for higher molecular weight proteins. The gel band intensities were profiled and the region with observable differences was identified.

### Capillary-liquid chromatography nanospray tandem mass spectrometry

Capillary-liquid chromatography nanospray tandem mass spectrometry (nano-LC/MS/MS) was performed on a Thermo Finnigan LTQ mass spectrometer. The LC system was an UltiMate™ Plus system from LC-Packings A Dionex Co. with a Famos autosampler and Switchos column switcher (Sunnyvale, CA). Solvent A was 50 mM acetic acid in water, and the solvent B was acetonitrile. Each sample (5 μl; tryptic peptides from the in-gel or solution enzymatic digestion) was injected on to the trapping column (LC-Packings A Dionex Co., Sunnyvale, CA) and washed with solvent A then loaded to a 5 cm 75 μm i.d. ProteoPep II C18 column (New Objective Inc., Woburn, MA) packed directly in the nanospray tip. Peptides were eluted directly off the column into the LTQ system using a gradient of 2%–80% B over 30 min with a flow rate of 300 nl/min. The scan sequence of the mass spectrometer was based on the TopTen™ method; a full scan is acquired and a subsequent MS/MS scan is acquired in consecutive instrument scans of the 10 most abundant peaks in the spectrum. Dynamic exclusion was used to exclude multiple MS/MS of the same peptide.

### Bioinformatics

Sequence information from the MS/MS data was processed by converting the raw data files into a merged file (.mgf) using MGF creator (merge.pl, a Perl script). The resulting .mgf files were searched using Mascot Daemon by Matrix Science (Boston, MA). Data processing was performed following published proteomic guidelines [49]. The mass accuracy of the precursor ions was set to 2.0 Da, and the fragment mass accuracy was set to 0.5 Da. Considered modifications (variable) were methionine oxidation and cysteine carbamidomethylation. Protein identifications were checked manually. The Mowse (molecular weight search) score [50] is a probability-based scoring algorithm for peptide matching and protein identification, and only Mowse scores of 80 or higher were accepted with a minimum of two unique peptides from one protein having a *-b* or *-y* ion sequence tag of five residues or better.

Protein classifications were determined using Protein ANalysis THrough Evolutionary Relationships (PANTHER), the classification of genes and proteins. PANTHER classifies genes by their functions and categorizes them by molecular function and biologic purposes. The protein function and location were determined from the Human Protein Reference Database.

**Figure 4:**
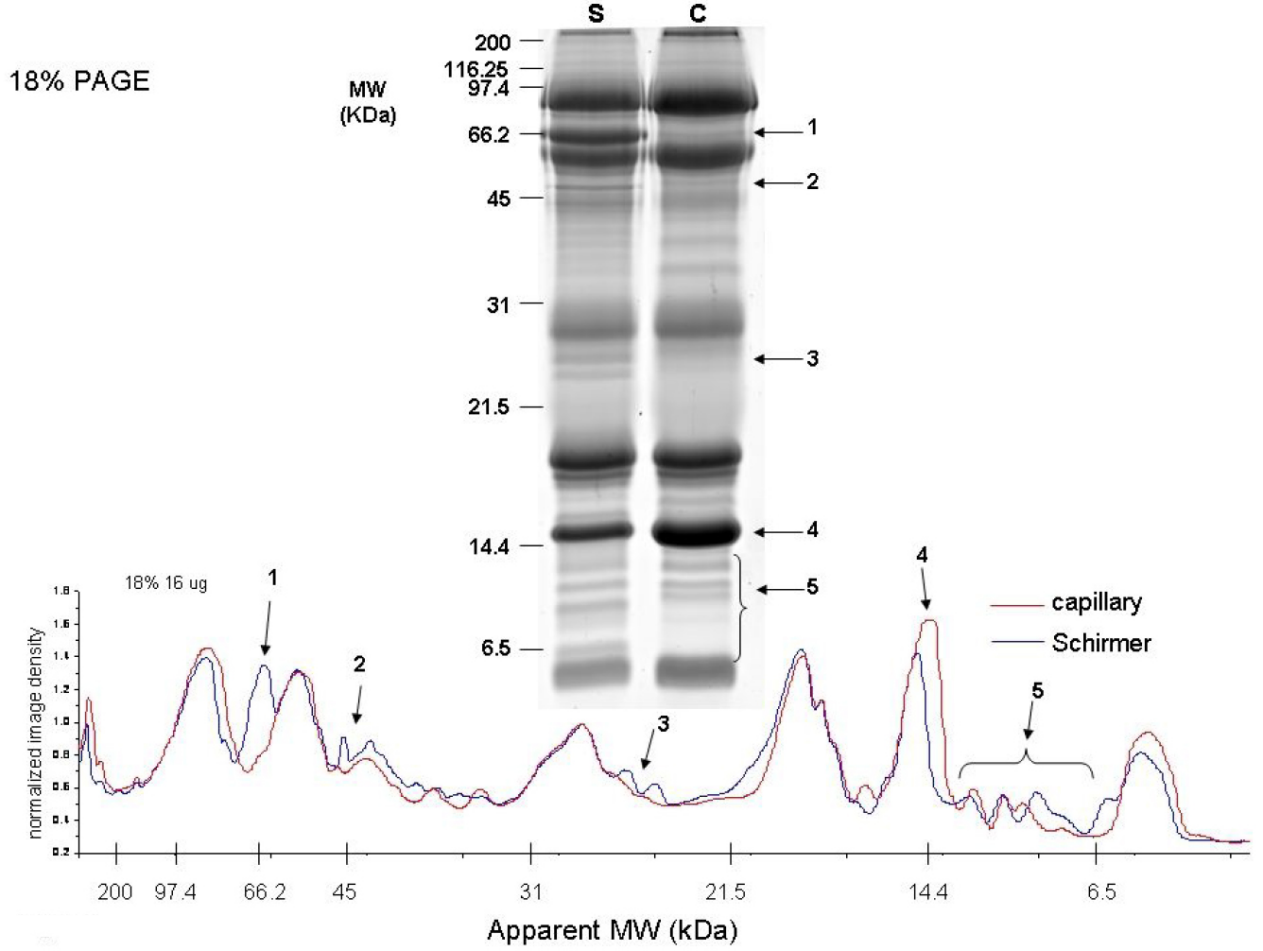
18% 1D-SDS–PAGE of coomassie stained proteins optimized for lower molecular weight proteins. The gel band intensities were profiled and the region with observable differences was identified.

### MudPIT

Ammonium sulfate (70%) was used to fractionate the tear samples with the intent to detect the lower abundant proteins in the samples. Four samples were examined by MudPIT: (1) Capillary-collected, ammonium sulfate precipitation; (2) Capillary-collected, ammonium sulfate supernatant; (3) Schirmer-collected, ammonium sulfate precipitation; and (4) Schirmer-collected, ammonium sulfate supernatant.

Precipitated proteins were resuspended in 20 μl of 70% saturated ammonium sulfate (SAS) in water. Samples were incubated for 1 h at room temperature, and precipitates were pelleted by centrifugation. Supernatants (70% SAS soluble fractions) were moved to new tubes, and pellets (70% SAS insoluble fractions) were resuspended in 20 μl of 70% SAS. Water (300 μl) and 100% trichloroacetic acid (100 μl) were added to all samples, and proteins were precipitated at 4 °C for 1 h. The precipitated protein was pelleted by centrifugation, the supernatant was discarded, and the pellets were then washed with acetone and air dried.

Pre-fractionated proteins from Schirmer and capillary strips were digested with trypsin in solution. Five micrograms of the 70% SAS soluble fractions and 10 μg of the SAS insoluble fractions were brought to a volume of 5 μl each in solubilization buffer (8 M urea, 1% CHAPS). The samples were then reduced with dithiothreitol (2.5 μl of 5 mg/ml dithiothreitol in 100 mM ammonium bicarbonate) at 37 °C for 1 h. Iodoacetamide (2.5 μl of 15 mg/ml iodoacetamide in 100 mM ammonium bicarbonate) was then added to alkylate the cysteines, and solutions were incubated for 1 h at room temperature in the dark. Sequencing grade trypsin (5 μl; Promega) prepared in water (25 ng trypsin per 1 μg of protein sample) and 5 μl of 100 mM ammonium bicarbonate were added, and samples were digested at 37 °C for 5 h in a heated water bath.

**Figure 5:**
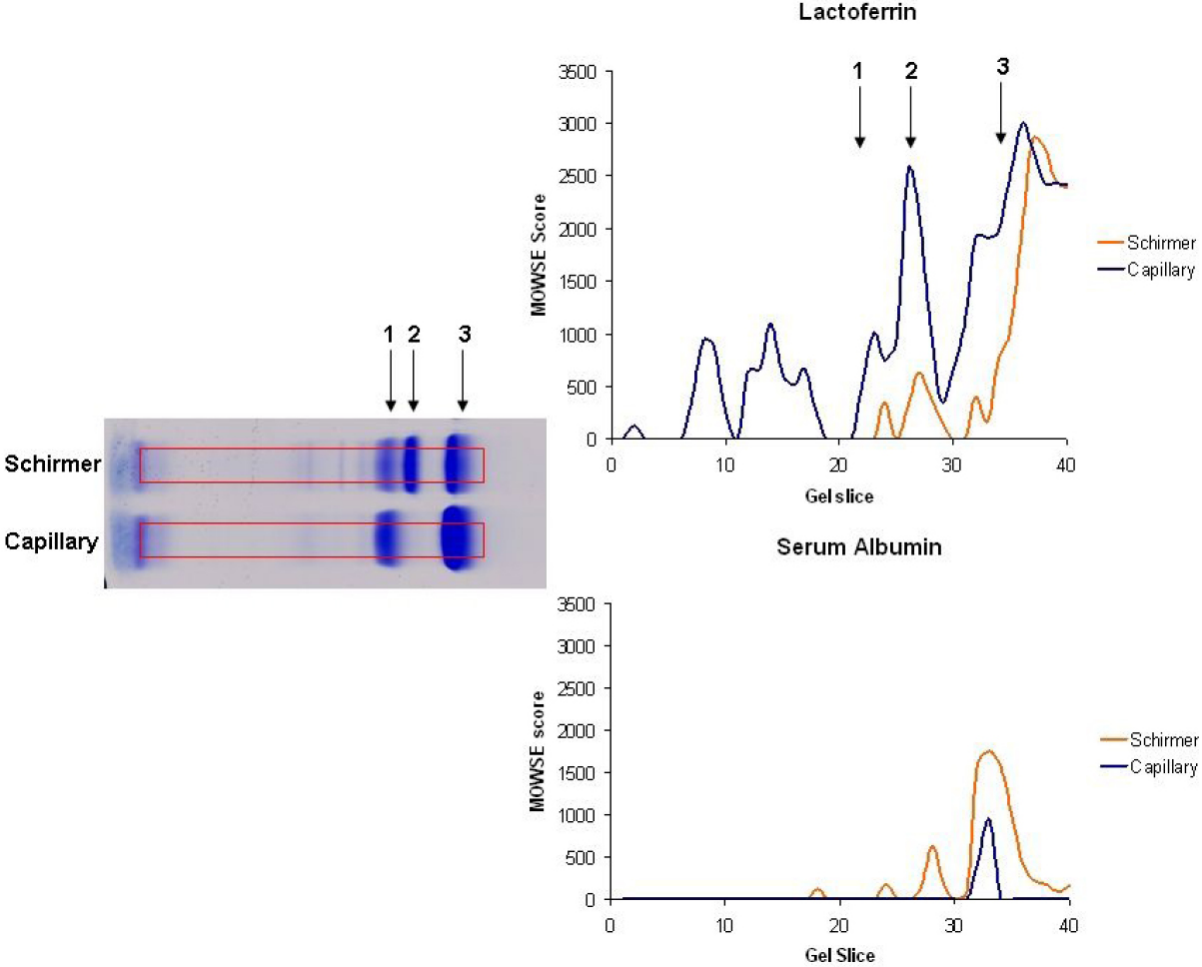
1D-SDS–PAGE of coomassie stained lactoferrin and serum albumin. Shown is a graph of gel slice versus Mowse score of lactoferrin and serum albumin to determine what protein is the dominating factor from the gel band.

The same LC-MS system described above was used for 2D LC-MS/MS. Each sample (5 μl) was injected on a strong cation exchange (SCX) column (10 cm, 300 μm i.d. Poros 10S; LC Packings Sunnyvale, CA) for the first dimension. Peptides initially not retained on the SCX column were eluted to a C18 trapping column (LC-Packings A Dionex Co., Sunnyvale, CA) and washed with 50 mM acetic acid to desalt the peptides. The peptides were eluted off of the trapping column onto the C18 column into the LTQ system for separation as described above. Ammonium acetate injections (salt plugs) were used to elute peptides stepwise from the SCX and then onto the C18 as described above. Injections (20 μl) of 10, 25, 50, 100, 200, 500, 1000 mM ammonium acetate were used.

## Results

### Quantitation

The total protein amount collected by capillary averaged 7.0 ± 1.8 μg/μl (around 35 μg per 5 μl of tears). Typical amounts of protein collected by Schirmer Strip were about 120 μg of total protein per Schirmer strip. It is difficult to ascertain similar protein concentrations on total protein quantities from a Schirmer strip since the volume collected cannot be measured. However, based on qualitative comparison, there was far more total protein collected by the Schirmer strip method compared with the capillary collection method.

**Figure 6:**
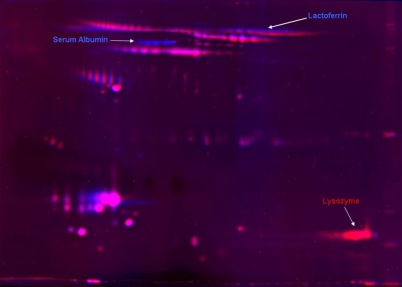
2D-SDS–PAGE. A seven centimeter 2D-SDS–PAGE of capillary collected and Schirmer extracted tear proteins stained with SYPRO Ruby and overlaid to show the contrasting proteins observed between the two collection methods. The red channel represents the image for capillary collected tears and the blue channel represents the image from the tear proteins extracted from a Schirmer strip.

### 1D-SDS–PAGE and LC-MS/MS

To examine the difference in amount of protein content between capillary-collected and Schirmer-collected tear film, samples were initially analyzed by 1D-SDS–PAGE gradient gel and shown in Figure 2. Total protein amounts were measured based on equal load amounts of protein (20 μg) in each lane (despite the difference in protein quantities associated with the two methods). Loading equal amounts ensures that the differences noted in the gel patterns are from the differences in the presence/absence of proteins from the collection methods rather than one method simply having more protein than the other. The observed bands were sliced into 11 regions for each collection method (e.g., lane) with a total of 22 bands. Lane 3 (Schirmer strip) has several bands that are more visible than Lane 2 (capillary) all of which seem to fall in the 30–66 kDa range (e.g., bands 2, 4, 5, and 6).

The proteins from 22 corresponding bands were identified by LC-MS/MS. Table 1 is a representative table of the protein identification data from band 4 including ascension number, Mowse score, number of peptides, and sequence coverage. All subsequent protein identifications were tabulated in this manner but for brevity, are not included in future results presented here. Instead, a summary of all proteins identified through the different proteomic methods, the protein function and location (determined from the Human Protein Reference Database) is listed in Appendix 1.

**Figure 7:**
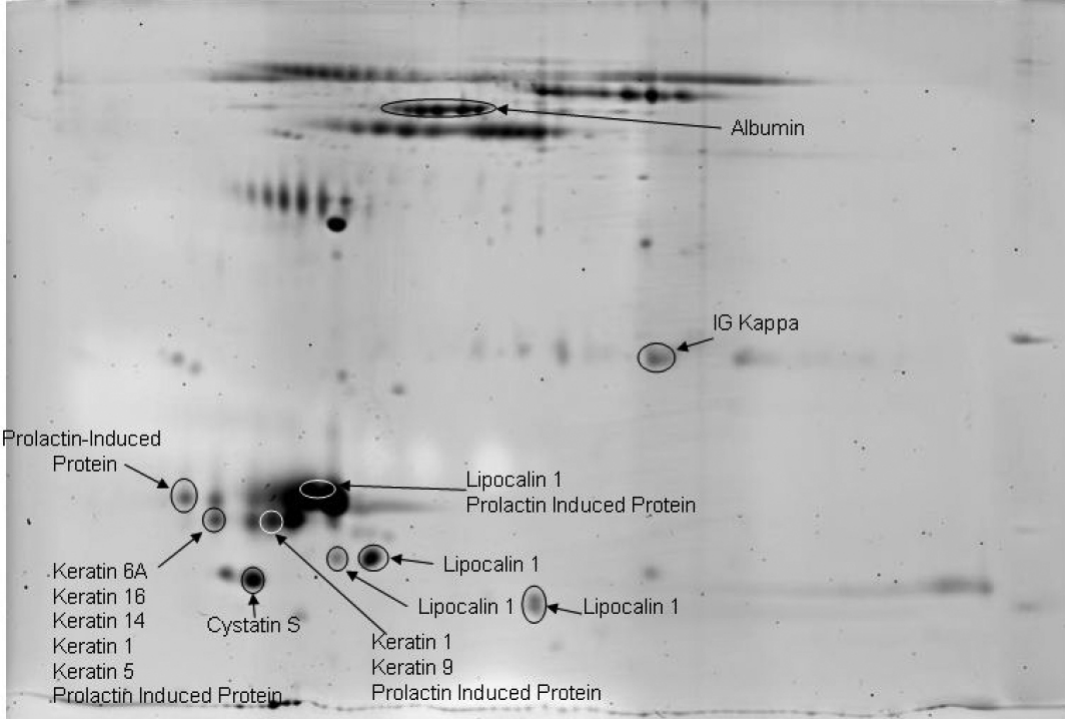
Eighteen-centimeter 2D-SDS–PAGE stained with SyproRuby of Schirmer strip-collected tears. The gel is labeled with subsequent protein identifications by nano-LC/MS/MS.

From the 11 bands associated with the capillary collection, a total of 40 distinct/unique proteins were identified. Several proteins were observed multiple times at different molecular weight regions of the gel. For example, basic proline rich protein (a lacrimal gland-associated protein) has a molecular weight of 22.8 kDa, although it was also observed in Band 2C at approximately 65 kDa. This likely represents posttranslational modifications or the formation of protein homopolymers (e.g., dimers, trimers, and multimers of a protein) of lower molecular weight proteins. It could also represent protein complexes that were not denatured. There are also higher molecular weight proteins observed at lower molecular weight regions in the gel (e.g., lactoferrin is observed throughout the gel). This could be from protein degradation occurring from storage or tear proteases or from sample carryover between analyses especially for high abundant proteins like lactoferrin.

From the 11 bands associated with the Schirmer strip collection method, 66 unique proteins were identified and are listed in Appendix 1. Band 2S (Lane 3, Figure 2) is quite prominent while it is much weaker in the corresponding band (Band 2C) from the capillary collected tears (Lane 2). As shown in Appendix 1, several well known cellular proteins including heat shock protein 70–1 (HSP70–1; Band 2S), keratin proteins, and a series of S-100 calcium binding proteins are observed from the Schirmer collection but not from the corresponding capillary. Similarly, proteins identified in bands 7S, 8S, 9S, 10S, and 11S (from the Schirmer strips) are quite different from the corresponding bands associated with capillary collection (Bands 7C, 8C, 9C 10C, and 11C).

The dynamic range of protein molecular weight found in the tear film is large; therefore, the 1D profiles of the tear film were analyzed using different percentages of polyacrylamide. Figure 3 shows the SYPRO Ruby-stained 10% SDS–PAGE (optimized for higher molecular weight proteins) with 16.7 μg total protein each from Schirmer and capillary collections and the corresponding image intensity profiles from these gels. Regions identified with the most significant differences between the two collection methods are labeled 1–4 on both the gel and corresponding intensity profile graph. Similarly, Figure 4 is an 18% SDS–PAGE (optimized for lower molecular weight proteins) of 16.7 μg total protein from the Schirmer and capillary collection methods and the corresponding image intensity profiles from these gels. Notable differences are labeled 1–5 on both the gel image and the corresponding image intensity profiles. A gel splicer that cuts the gel into 40 equal bands was used to attempt to identify every protein in the entire lane, and the data from the resulting protein identification are listed in Appendix 1. Thirteen additional proteins from the Schirmer collection method and seven additional proteins from the capillary collection method were detected using the gel splicer.

**Figure 8:**
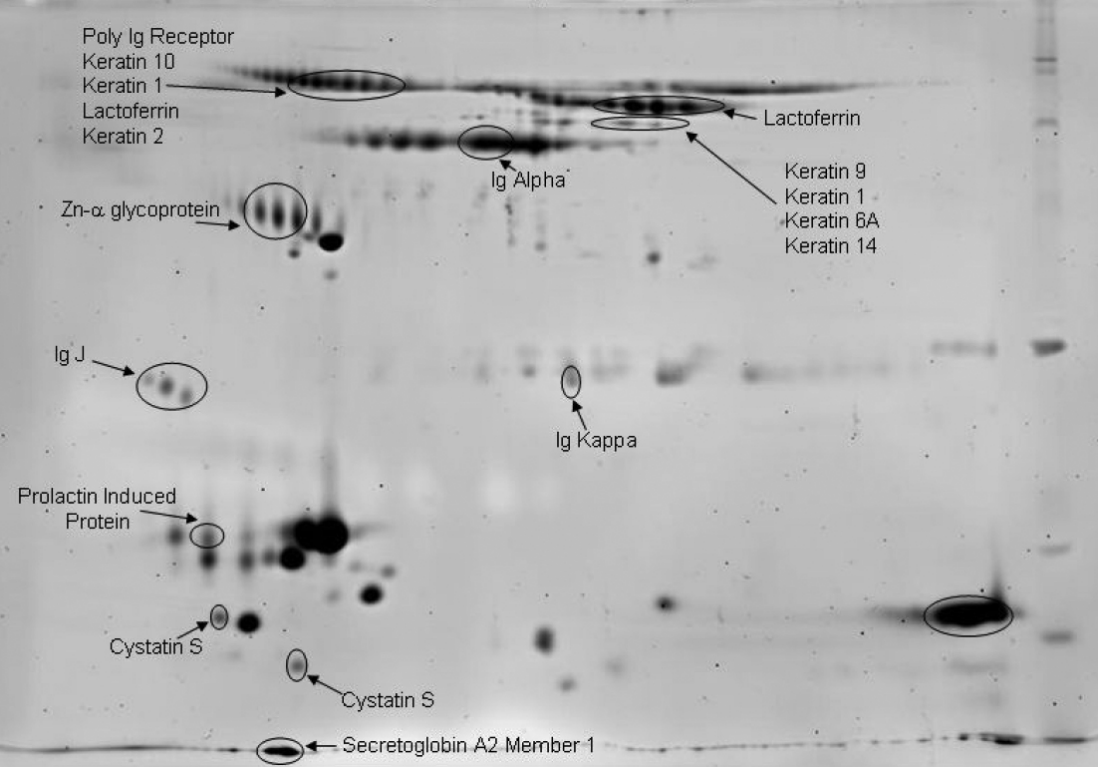
Eighteen-centimeter 2D-SDS–PAGE stained with SyproRuby of capillary-collected tears. The gel is labeled with subsequent protein identifications by nano-LC/MS/MS.

The sequence coverage observed in the mass spectrometry experiments reflects the amount of the protein. Highly abundant proteins yield high sequence coverage while low abundant proteins yield low sequence coverage (assuming that digestion is complete, the protein has a good digestion pattern, and the peptides do not suffer from unusually low ionization efficiencies). The protein score is derived from the individual ion scores and, using the same logic, the higher the protein score, the higher the abundance of a particular protein in the sample. As the gel was sliced into equal amounts and the protein score (Mowse) is loosely correlated to protein abundance in the sample, a plot of the gel slice versus Mowse score for a single protein can be generated. The purpose of this experiment was to plot what specific protein contributes to the visual band. Figure 5 shows the gel slice plotted against the Mowse scores for lactoferrin and serum albumin (obtained from the protein identifications from the 10% gel). The Schirmer method has a relatively low presence of lactoferrin in the regions marked 1 and 2 whereas the capillary has a high presence of lactoferrin in the same region. The opposite is true of serum albumin where it is observed a higher presence of serum albumin associated with the Schirmer method than the capillary collected tears.

### 2D-SDS–PAGE analyses and LC-MS/MS

Differences in protein patterns between capillary- and Schirmer-collected tears were examined on a 7-cm (mini) 2D-SDS–PAGE (Figure 6 with SYPRO Ruby staining with the capillary and Schirmer method gels overlaid). Several regions in the 2D analysis show significant differences in protein patterns as was also observed with the 1D gel band patterns. One noteworthy region is observed in the high molecular weight region as an intense blue band, indicating a predominance of proteins extracted from the Schirmer method. LC-MS/MS protein identification of this region indicated serum albumin. The streak observed in the blue and red channel, which is also in the high molecular weight region, corresponds to lactoferrin. These results agree with the results from the 1D analysis Mowse plots that there are differences in lactoferrin and serum albumin between the collection methods. Finally, there is an intense red channel protein identified as lysozyme. While it is well known that lysozyme is a highly abundant protein in the tear, it appears to not be efficiently recovered from the Schirmer collection method.

Figure 7 and Figure 8 are the individual SyproRuby stained gels labeled with the protein identifications. Similar to other published work [10,45,51,52], as many as 500 protein spots are observed in the 2D gel. 2D electrophoresis is not the most efficient way to identify all the proteins in a complex mixture of proteins. Rather, it is better suited to examine protein pattern changes between two samples. In this case, 2D gel electrophoresis was mainly used to examine pattern changes observed between capillary- and Schirmer strip-collected tear films. Protein identifications were conducted on 58 selected spots that were cored, digested, and analyzed by nano LC-MS/MS. A total of 31 unique proteins were identified, and 27 of the 58 spots matched proteins identified from other cores in other regions of the gel, similar to previous results (e.g., the multiple spots along the 80 kDa region are predicted to be glycosylated lactoferrin).

**Table 2:** Summary of proteins observed by MudPIT after fractionation with ammonium sulfate.

**Protein from capillary**	**Precipitant**	**Supernatant**
Lactoferrin	X	X
Lipocalin 1	X	X
Poly Ig Receptor	X	X
Ig A1 Bur	X	X
Zn-alpha2-glycoprotein	X	X
Proline Rich 4	X	X
Cystatin S	X	
Ig Alpha 1	X	X
Ig Lambda	X	
Ig Alpha 2	X	X
Secretoglobin, family 2A Member 1	X	X
Protein Len, Bence-Jones	X	
Prolactin Induced Protein	X	X
Protein Rei, Bence-Jones	X	
Ig Kappa	X	
Lysozyme	X	X
Lacritin	X	X
Basic proline rich protein	X	
DMBT1	X	
Ig J	X	
Keratin 1	X	X
Lipophilin A	X	X
Haptoglobin	X	
CTBP2 Protein	X	
Transcoalbumin I	X	
Keratin 9		X
Keratin 2a		X
**Protein from Schirmer**	**Precipitant**	**Supernatant**
Lactoferrin	X	X
Lipocalin 1	X	X
Poly Ig Receptor	X	
Zn-alpha2-glycoprotein	X	
Proline Rich 4	X	X
Ig A1 Bur	X	X
Prolactin Induced Protein	X	X
Ig Alpha 2	X	X
Ig Alpha 1	X	
Secretoglobin, family 2A Member 1	X	
Protein Len, Bence-Jones	X	
Protein Rei, Bence-Jones	X	
Lysozyme	X	X
Ig Kappa	X	X
Cystatin S	X	X
Lactritin	X	X
Basic proline rich protein	X	
Ig J	X	
Serum Albumin	X	X
DMBT1	X	
HRPE773	X	
Lipophilin A	X	
Haptoglobin	X	
Ig Gamma	X	
Keratin 1	X	X
actin, beta	X	X
Keratin 9		X

Ammonium sulfate precipitation was used to fractionate and remove the high abundant proteins from less abundant proteins. This Table summarizes the proteins identified from the various fraction and collection methods. In both the capillary-collected tears and Schirmer-collected tears, the precipitant fraction contained the most proteins and the supernatant contained what are known to be the highly abundant proteins in the tear.

### MudPIT

Finally, capillary-collected tear samples and proteins extracted from the Schirmer collection method were analyzed by digesting the sample without prior separation by SDS–PAGE before nano LC-MS/MS (i.e., a MudPIT proteomic approach) [5]. Ammonium sulfate precipitation was used to fractionate and remove the high abundant proteins from less abundant proteins. Table 2 summarizes the proteins identified from the various fraction and collection methods. In both the capillary-collected tears and Schirmer-collected tears, the precipitant fraction contained the most proteins and the supernatant contained what are known to be the highly abundant proteins in the tear. While the fractionation did appear to distribute the protein content between soluble and insoluble fractions, only 28 unique proteins were identified this way. It is interesting to note that, based on this analysis method, there is virtually no difference in the protein identifications between capillary-collected tears and proteins extracted from the Schirmer strip.

**Figure 9:**
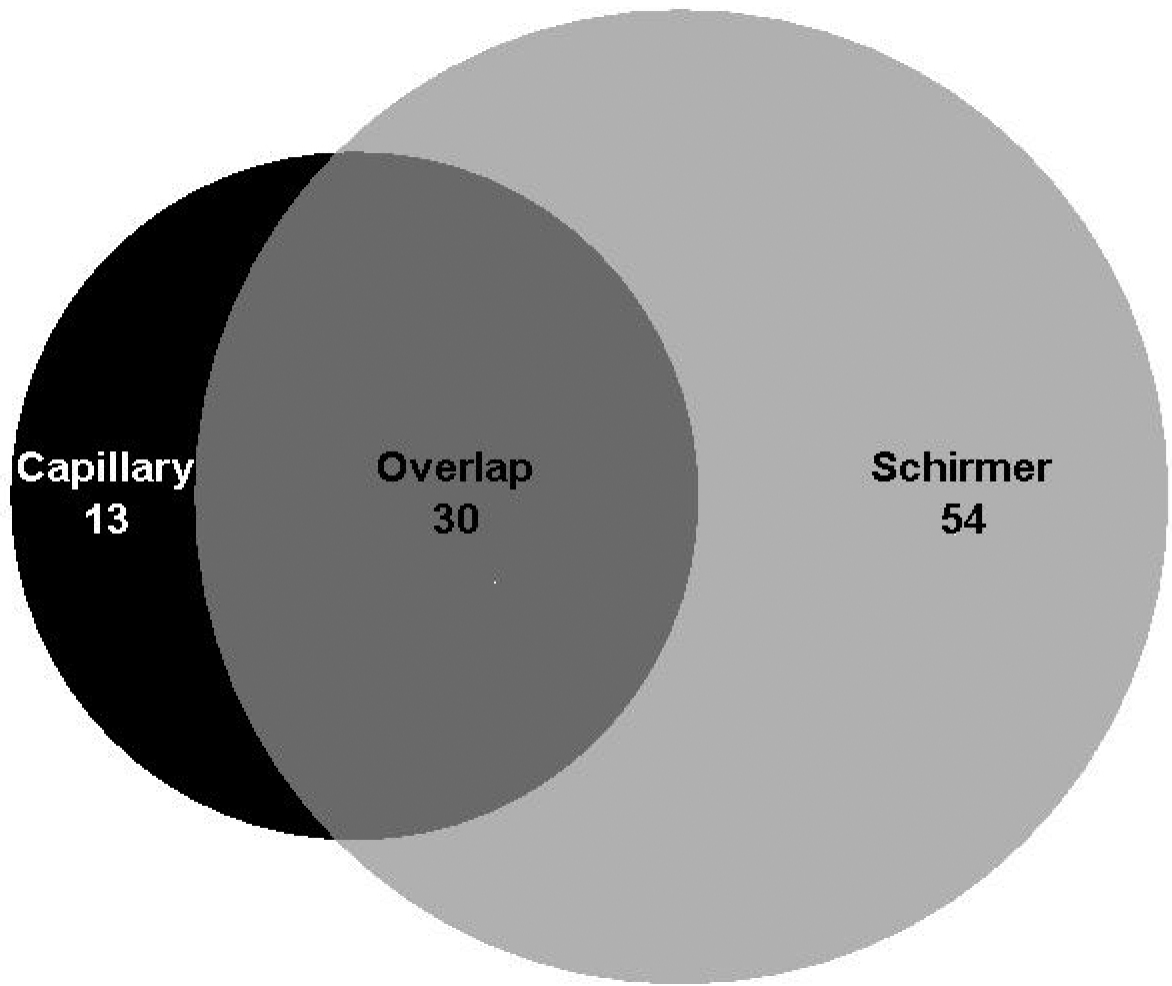
Venn diagram comparing the distribution envelope of proteins that were collected by capillary versus by Schirmer strip. The proteins were identified using GeLC-MS/MS and MudPIT.

**Figure 10:**
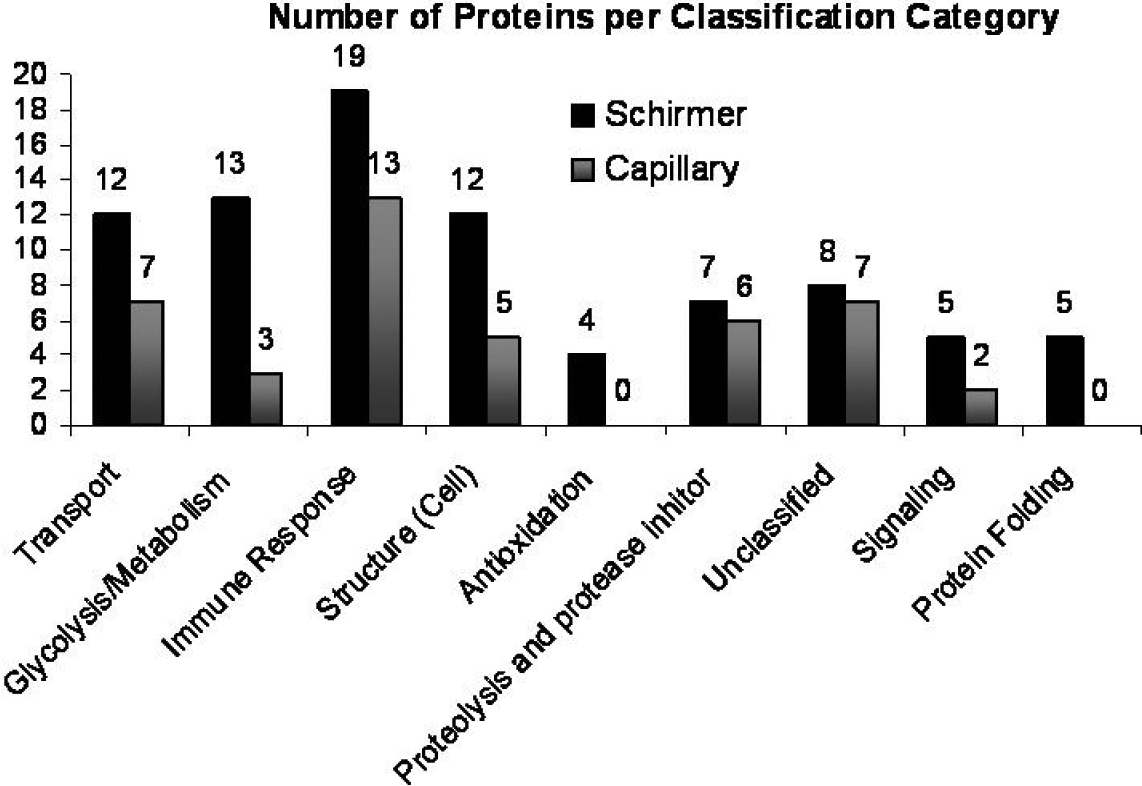
Graph representing number of proteins observed in each classification of protein function. The protein functions are described as transport, metabolism, immune response, structure, antioxidation, protease inhibitor, unclassified signaling, and protein folding.

## Discussion

The analysis of the tear film has the “high abundance” problem similar to analyses of serum, whereby a handful of highly abundant proteins (such as lactoferrin, lysozyme, and albumin) masks the lower abundant proteins [53-56]. As shown in other proteomic works associated with the plasma, multiple proteomic approaches are required to reveal unique proteins while avoiding sequence or splice variants and cleavage products relative to capturing the entire proteome. In total, it appears that 1,175 proteins were identified in the plasma by these multiple approaches, but only 46 were identified by all four methods used [57]. While mass spectrometry-based proteomics is very sensitive (nanogram – picogram sensitivity), it does suffer from a limited dynamic range (refers to the range of values that can be measured) for biologic fluids, which are extremely complex and have a large dynamic range in protein concentrations. For example, the protein epidermal growth factor (EGF) was not identified in this study nor the de Souza study despite the fact that it is known from immunological methods [58,59]. Likewise, various cytokines and matrix metalloproteases were also not found in this study. Although these were normal tear film samples, their absence is not necessarily surprising [60-63]. Therefore, proteins present in very low levels are not detected because they are masked by the presence of very high concentrated proteins. The purpose of this study was to further develop and understand the normal human tear film proteome similar to recent scientific activity as it relates to the human plasma [57]. Appendix 1 sums all the proteins identified using the methods described in this paper with the 30 proteins described as the core tear proteome highlighted in gray. Approximately four times as many proteins were identified from 1D SDS–PAGE followed by in-gel digestions compared to direct digestion of the proteins using the MudPIT approach (97 from gels and approximately 28 from MudPIT). This is very similar to the results observed by Zhou and coworkers [12], although a total of 97 unique proteins were identified, which is far less than the 491 proteins identified by the de Souza [6] paper. The study by de Souza used a high resolution mass spectrometer and MS^3^ capabilities allowing for highly reliable protein identification from only a single peptide, whereas the low resolution ion-traps used in this study requires a minimum of two peptides to reliably identify proteins. However, there were several of the same proteins observed in this work that were also observed by Zhou but were excluded in our study as only a single peptide was sequenced.

The most proteins (n=97) identified were from the 1D-SDS–PAGE and nano-LC-MS/MS approach followed by the 2D-SDS–PAGE and nano-LC-MS/MS approach (n=32) and lastly, the MudPIT approach (n=28) which is associated with capillary collection discussed below. It is possible that more unique proteins could have been identified from the 2D electrophoresis. However, methods like MudPIT and protein identification from 1D gels are a more efficient way to detect proteins from a complex mixture. There were 30 proteins identified by all three methods (listed as the first 30 proteins and shaded in gray in Appendix 1), and this likely represents the core of the tear film proteome (i.e., the most abundant proteins). The MudPIT approach (n=28 proteins) seems to have identified mainly the highly abundant proteins in the tears (e.g., lactoferrin, lipocalin, etc.). Perhaps by using a similar approach as in serum proteomics where the high abundant serum proteins are removed before MudPIT analysis using affinity removal, columns would lend itself to a better examination of the lower abundant proteins via this method. The 2D-SDS–PAGE provided better insight to the overall pattern changes of proteins than the other methods, although it is not practical to core all the proteins observed in a 2D gel for subsequent identification. The first reason is that the amount of protein required for a large 2D gel can limit a proteomic project to pooled samples, thus potentially limiting large clinical studies of individual patients in terms of individual analyses. More specifically, a single tear sample of a healthy person contains roughly 10 μg of protein, and a recommended protein load for protein identification from a large format 2D gel is approximately 300 μg of total protein. The second reason is that 2D SDS–PAGE followed by LC-MS/MS is more practical when choosing certain protein spots that are observed to change with disease, environmental challenge, or treatment with a drug and is not necessarily meant for complete protein identification of the total proteome. The 2D-SDS–PAGE followed by LC-MS/MS approach will be more valuable when looking for up and or down regulation of proteins.

A secondary goal of this research was to compare methods of tear film collection (i.e., capillary collection versus Schirmer collection). A Venn diagram in Figure 9 shows the overlap of proteins identified between the two collection methods. There were 84 proteins identified from protein associated with the Schirmer method and 43 identified from the capillary method. Only 30 total proteins identified overlapped between the two collection methods. We propose that this difference arises through the Schirmer strip’s interaction with the epithelium of the ocular surface (whereas the capillary does not). To help examine this hypothesis, analysis of the various classifications/functions of the proteins identified were grouped based on their general function as follows transport, metabolism, immune response, structure, antioxidation, protease inhibitors, unclassified, cell signaling, and protein folding. Figure 10 is a graph of the number of proteins found in each classification group compared by collection method. There are several cellular proteins (i.e., not secreted) observed from the Schirmer method that were not found in tear film collected by capillary such as the S100 calcium binding series of proteins. Interestingly, serum albumin was detected at much higher levels in the proteins associated with the Schirmer collection method. As shown in Figure 10, no proteins classified as antioxidants were found in the capillary-collected tears, but four are found in proteins extracted from Schirmer. Similarly, five proteins classified as protein-folding proteins are found in the Schirmer-collected tears whereas none were detected in the capillary-collected tears. There are also more proteins in the metabolism and cell structure classifications from Schirmer-collected tears, and proteins classified as transport and immune response proteins have notable differences between the two collection methods. Lastly, proteins classified as structure-related protease inhibitors and those that could not be classified show similar levels between the two collection methods. Overall, these results suggest that the tear film collection method does impact the proteins present in the sample and that care should be exercised in choosing a tear collection method to best correlate to the experiment being conducted or the hypothesis that is being tested.

## Appendix 1. Summary of all proteins observed by all of the various proteomic approaches and collection methods.

To access the data, click or select the words “Appendix 1”. This will initiate the download of a compressed (zip) archive that contains the file. This file should be uncompressed with an appropriate program (the particular program will depend on your operating system).
